# TGF‐β1 promotes gap junctions formation in chondrocytes via Smad3/Smad4 signalling

**DOI:** 10.1111/cpr.12544

**Published:** 2018-11-15

**Authors:** Qingxuan Wang, Chenchen Zhou, Xiaobing Li, Linyi Cai, Jing Zou, Demao Zhang, Jing Xie, Wenli Lai

**Affiliations:** ^1^ State Key Laboratory of Oral Diseases, Orthodontics Department, West China Hospital of Stomatology Sichuan University Chengdu China

**Keywords:** chondrocyte, connexin43, GJIC, Smad3, Smad4, TGF‐β1

## Abstract

**Objectives:**

Connexin‐mediated functional gap junction intercellular communication (GJIC) has a vital role in development, homeostasis and pathology. Transforming growth factor‐β1 (TGF‐β1), as one of the most vital factors in chondrocytes, promotes cartilage precursor cell differentiation and chondrocyte proliferation, migration and metabolism. However, how TGF‐β1 mediates GJIC in chondrocytes remains unclear. This study aims to determine the influence of TGF‐β1 on GJIC in mouse chondrocytes and its underlying mechanism.

**Methods:**

qPCR and mRNA microarray were used to verify the expression of genes in the TGF‐β and connexin families in cartilage and chondrocytes. A scrape loading/dye transfer assay was performed to explore GJIC. Western blot analysis was used to detect connexin43 (Cx43) and Smad signalling components. Immunofluorescence staining was performed to characterize protein distribution.

**Results:**

The TGF‐β1 mRNA was the highest expressed member of the TGFβ super family in cartilage. TGF‐β1 promoted functional GJIC through increased expression of Cx43. TGF‐β1‐mediated GJIC required the participation of TGF‐β type I receptor. TGF‐β1 activated Smad3 and Smad4 signalling to facilitate their nuclear translocation. The Smad3 and Smad4 signalling proteins bound to the promoter of *Gja1* and thus initiated Cx43 gene expression.

**Conclusions:**

For the first time, these results revealed a vital role of TGF‐β1 in cell‐cell communication in chondrocytes via gap junction formation. We describe the regulatory mechanism, the involvement of TGF‐β type I receptor and the nuclear translocation of Smad3/4.

## 1 INTRODUCTION

Articular cartilage is a highly specialized connective tissue that wraps the end of bones to form low‐friction surfaces essential for painless movement of joints. In contrast to bone and other connective tissues, cartilage is an avascular tissue composed of extracellular matrix (ECM), which primarily consists of collagen and proteoglycans. Chondrocyte are the only cells found in healthy cartilage and have roles in mechanical response and cartilaginous matrix maintenance, formation and repair.^1^ Proper cell‐to‐cell and cell‐to‐matrix communication is necessary for the maintenance of tissues and regulates multiple cellular pathways, including proliferation, differentiation and cell death. Intercellular communication travels through gap junction channels, and cell‐ECM communication travels through hemichannels, which are single‐membrane diffusion channels that allow passage of signalling molecules smaller than 1.2 kDa, such as metabolites, ATP, ions, second messengers and cyclic nucleotides.^2^, ^3^

Gap junctions are formed by connexin subunits. To date, there are at least 21 connexin family members in human and 20 in mice.^4^ The expression patterns of connexins differ according to cell type and function. Among the connexin family members, connexin43 (Cx43), encoded by the *Gja1* gene, is the main component of gap junctions in human tissue. It is widely expressed in bone cell types and is most abundant in osteoblastic cells, which are characterized by their distinct function in the skeletal maturation stage.^5^, ^6^ Cx43 has a substantial effect on chondrocyte mechanotransduction and cell cycle.^7^, ^8^ Accumulating evidence suggests that Cx43 hemichannels mediate homeostasis in cartilage and that impaired Cx43 hemichannels contribute to the aetiology of joint diseases.^9^

The TGF‐β super family is a large collection of secreted and structurally related proteins that are responsible for numerous cellular processes and have significant effects on the development and homeostasis in mammals.^10^, ^11^ This super family contains 33 members in the mammalian genome, which include the TGF‐β subfamily, the activins and inhibins, the bone morphogenetic proteins (BMPs) and the growth and differentiation factors (GDFs).^12^ Transforming growth factor‐β1 (TGF‐β1), a critical chondrocyte factor, plays a vital role in regulating cell proliferation, migration, differentiation, ECM formation and other developmental processes.^13^, ^14^, ^15^ However, whether TGF‐β1 induces cell‐cell communication in chondrocytes needs to be further investigated.

In the current study, we used recombinant TGF‐β1 to establish the relationship between TGF‐β1 and gap junctions in chondrocytes. To investigate the effect of TGF‐β1 on cell‐cell communication, changes in cell migration and proliferation, gap junction formation and its regulator Cx43 were detected. Furthermore, we provided direct evidence that the TGF‐β type I receptor and Smad3/4 signalling factors are involved in TGF‐β1‐mediated induction of Cx43.

## 2 METHODS AND MATERIALS

### 2.1 Preparation of tissues and cells

Animal cells and tissues used in the present study were obtained according to ethical principles, and all protocols were reviewed and approved by the Institutional Review Board (IRB, Institutional Review Board at the West China Hospital of Stomatology, No.WCHSIRB‐D‐2017‐029) at our hospital.

Articular cartilage tissue was collected from 8‐week‐old C57BL mice. Briefly, the articular cartilage was isolated, rinsed and immediately crushed into powder using liquid nitrogen. The fine residue was gathered for PCR assays. For chondrocytes, articular cartilage from newborn mice was shredded into small pieces and rinsed twice in 1 × PBS. Cartilage was trypsinized in 0.25% protease solution dissolved in Dulbecco's modified Eagle's medium (high‐glucose DMEM, 0.1 mmol/L non‐essential amino acids, 4 mmol/L l‐glutamine, 1% penicillin‐streptomycin solution, HyClone, Logan, UT, USA) for 30 minutes at 37°C and was transferred into 0.5% type II collagenase to digest overnight. Next, the type II collagenase‐treated solution was neutralized 1:1 (v/v) with 10% heat‐inactivated foetal bovine serum (FBS) in DMEM (high‐glucose DMEM, 0.1 mmol/L non‐essential amino acids, 4 mmol/L l‐glutamine, 1% penicillin‐streptomycin solution), and the suspension was centrifuged at 157 ***g*** (relative centrifugal force) for 5 minutes. The remaining chondrocytes were re‐suspended in 10% FBS DMEM with 1% penicillin‐streptomycin and then seeded into plates or T25 flasks and incubated in a 5% CO_2_ incubator at 37°C. Passages 1‐2 were used in the experiments.

### 2.2 RNA extraction and quantitative real‐time PCR

Cells were washed with cold PBS, and total RNA was extracted from chondrocytes (cells) and joint residue (tissue) with the RNeasyPlus Mini Kit (Qiagen, Shanghai, China) with a genomic DNA eliminator according to the manufacturer's instructions. The extracted RNA samples were dissolved in RNase‐free water and quantified by a spectrophotometer instrument measuring absorbance at 260 nm. After treatment with DNase I, RNA (0.5‐2 μg) was reverse‐transcribed to cDNA with the first‐strand cDNA synthesis kit (Mbi, Glen Burnie, MD, USA).

The sets of primers used to detect each gene in the TGF‐β super family are listed in Table 1. The BLAST programme was used to determine all primer sequences, and all samples were normalized to the housekeeping gene glyceraldehyde‐3‐phosphate dehydrogenase (GAPDH). Quantitative real‐time PCR (qPCR) was performed by amplifying a specific product with a reaction mixture containing cDNA, SYBR Green I PCR master mix (Takara, Tokyo, Japan) and primer pairs, and qPCR was run on an ABI 7300 instrument (Applied Biosystems, Shanghai, China) equipped with 96‐well optical reaction plates. The reaction conditions are as follows: 95°C for 10 minutes followed by 40 cycles of 95°C for 5 seconds and 60°C for 30 seconds. Each gene was assayed in triplicate. The fold change calculations were performed with the $2^{-\Delta \Delta C_{t}}$ method.

**Table 1:** Sequences of primer pairs of housekeeping and TGF‐β super family genes in chondrocytes for qPCR

Genes	Forward sequence	Reverse sequence	Product size (bp)
GAPDH	5′‐GGGTCCCAGCTTAGGTTCATC‐3′	5′‐AATCCGTTCACACCGACCTT‐3′	87
TGFβ1	5′‐CACTCCCGTGGCTTCTAGTG‐3′	5′‐CTTCGATGCGCTTCCGTTTC‐3′	102
TGFβ2	5′‐AAAATCGACATGCCGTCCCA‐3′	5′‐CAAGGTACCCACAGAGCACC‐3′	118
TGFβ3	5′‐CCTCAGGCTTTGGGATCTGG‐3′	5′‐TCATGTGTGAGCCCAGGAAC‐3′	114
BMP1	5′‐AGGGCGGCGAGAAAAGAAA‐3′	5′‐CTTCCCGTCCCGTTTCCTG‐3′	115
BMP2	5′‐CCGCTGTCTTCTAGTGTTGCT‐3′	5′‐TCTCTGCTTCAGGCCAAACAT‐3′	181
BMP3	5′‐GGTCGAACCTCGGAACTGTG‐3′	5′‐TCCTCTACCCCGTGCAAAAA‐3′	198
BMP3b	5′‐TCCCCATGCCCAAGATTGTC‐3′	5′‐GGTACACCTTCAGAACCGCA‐3′	171
BMP4	5′‐CAGGAACCAATGAGACACCAT‐3′	5′‐TTTTCTTCCCGGTCTCAGGT‐3′	119
BMP5	5′‐TTTCTGAGGAGTGGGGCTCT‐3′	5′‐GCTTGAAAGCTACAAGCGGG‐3′	121
BMP6	5′‐TAATGTTCGCCTCCCCCAAC‐3′	5′‐TCCCCTCCATTCGGATGTCT‐3′	178
BMP7	5′‐CCTATGGCCATGTCGCATCT‐3′	5′‐GCAGCCCAAGCTACTGAAGA‐3′	118
BMP8a	5′‐TGTGAGGGGGAGTGTGCTTT‐3′	5′‐CAGCAGGCTACTGTGGTACTGA‐3′	95
BMP8b	5′‐GTCCGGGACTCCTATGGCTA‐3′	5′‐ACCGGTGCTCGGGATCG‐3′	178
BMP9	5′‐TGCGCATGGTATGCCTAAGT‐3′	5′‐CCGCATACCCCAGTCATTGT‐3′	115
BMP10	5′‐AACGCCAAGGGGAACTACTG‐3′	5′‐TTCATACCCAGGAGGAGCGA‐3′	96
BMP11	5′‐GCACCCCTACCAAGATGTCC‐3′	5′‐CCACAACTTAGGAGCAGCCA‐3′	117
BMP12	5′‐GGTTCTGGCTTCAGGAACGG‐3′	5′‐CGCCGTTTCGTCTTGAGTTG‐3′	159
BMP13	5′‐CCCCAACTGGTTTGCTCTCT‐3′	5′‐GCATTCCGACCGTTTCCTTG‐3′	88
BMP14	5′‐GATCTGGCTGGGAGGTGTTC‐3′	5′‐AAGGCTTTCTCGTGGACCTG‐3′	160
BMP15	5′‐CCGGACCAAGCACTTACCTT‐3′	5′‐CGAAGAACACTCCGTCCCTT‐3′	135
GDF1	5′‐CAGCGGAGAATTGGATAGCA‐3′	5′‐GCAACATCTGCGCATAACTC‐3′	89
GDF3	5′‐TGGTAGTCGATGAGTGTGGG‐3′	5′‐TGTGTGTAATTGTGGGGCTCAT‐3′	158
GDF8	5′‐AGTACGACGTCCAGAGGGAT‐3′	5′‐TTGCCATCCGCTTGCATTAG‐3′	124
GDF9	5′‐TGAAGTCAGTCTTCCACACCT‐3′	5′‐CATCTCCTCGTGCCAGTCTT‐3′	88
GDF15	5′‐CCTCCATCTTCTATCTGAGCCTG‐3′	5′‐CCATGTCGCTTGTGTCCTTTC‐3′	200
Inhibinα	5′‐GCCAGTTCCTAAGCCCCTCT‐3′	5′‐CACTGGATCAGTCCCGCTTG‐3′	103
Inhibinβ	5′‐CACTTGCGGTCCTGAGTGAA‐3′	5′‐CAGTTTCGCCTAGTGTGGGT‐3′	162
Nodal	5′‐AGGGGGAGTGCTGAAATTGG‐3′	5′‐TTAGCTCCAGCAGGCAGAAC‐3′	114

BMPs, bone morphogenetic proteins; GAPDH, glyceraldehyde‐3‐phosphate dehydrogenase; GDF, growth and differentiation factors; qPCR, quantitative real‐time PCR; TGF‐β, Transforming growth factor‐β1.

### 2.3 Bioinformatics analysis

Gene information including the GeneBank ID and the promoter sequence (~2000 bp) upstream of the Gja1 transcriptional start site was acquired from the UCSC (https://www.genome.ucsc.edu /) and NCBI (https://www.ncbi.nlm.nih.gov/) resources. Transcription factor binding sites in the Gja1 promoter were predicted with the PROMO resource (https://alggen.lsi.upc.es/cgi-bin/promo_v3/promo/promoinit.cgi?dirDB=TF_8.3).

### 2.4 Immunofluorescence and confocal laser‐scanning microscopy

Primary chondrocytes were grown on dishes specified for confocal laser‐scanning microscopy (CLSM). After seeding, cells were starved using 2% FBS DMEM for 12 hours. After starvation, the culture medium was changed to 1% FBS DMEM with 1% penicillin‐streptomycin and TGF‐β1 (p04202; R&D Systems, Minneapolis, MN, USA). At specified experimental time points, the culture medium was discarded, and the cells were rinsed three times with PBS before fixation with 4% paraformaldehyde and permeabilization with 0.5% Triton X‐100 in 1% BSA. Cells were incubated with the following primary antibodies at 4°C overnight: Cx43 (ab11370; Abcam, Cambridge, UK), Smad3 (ab28379; Abcam) and Smad4 (ab40759; Abcam). A secondary antibody conjugated to AlexaFluor647 (ab150075; Abcam) was used for detection. All samples were counterstained with FITC‐phalloidin (Invitrogen, Carlsbad, CA, USA) overnight and DAPI (D9642; Sigma, St. Louis, MO, USA) for 10 minutes. Immunofluorescent images were captured using a CLSM (A1R MP+, Nikon, Tokyo, Japan and Olympus, FV3000, Japan).

### 2.5 Protein extraction and Western blot

Cell lysates were collected in lysis buffer containing PMSF (P7626; Sigma) protease inhibitor. The protein concentration was evaluated with a BCA Protein Assay.

Kit (Beyotime, Shanghai, China) and samples were mixed with Bio‐Rad Laemmli sample buffer (Bio‐Rad, Hercules, CA, USA) and boiled for 5 minutes at 100°C. Equal amounts of protein were separated by 10% SDS‐PAGE and transferred to polyvinylidene fluoride membranes. The membranes were blocked in Tris‐buffered saline containing 0.05% Tween 20 (TBST) and 5% non‐fat milk for 2 hours at room temperature. Next, the membranes were incubated overnight with relevant primary antibodies against Cx43, Smad3, Smad4 and β‐actin (sc‐47778; Santa Cruz Biotechnology, Cambridge, UK) as the internal control. After washing, membranes were incubated with corresponding anti‐mouse or anti‐rabbit secondary antibodies at room temperature for 2 hours. Immunoreactive blots were developed using the Super Signal reagents (Pierce, Rockford, IL, USA). ImageJ software (NIH, Bethesda, MD, USA) was used for densitometric analyses.

### 2.6 Cell Counting Kit‐8

Cells were seeded in a 96‐well plate at a density of 1.5 × 10^3^ cells per well and incubated in a 5% CO_2_ incubator at 37°C. Twelve hours after cell adherence, chondrocytes were treated with recombinant mouse TGF‐β1 at different concentrations for 24 hours in the presence or absence of repsox (ab14139, Abcam) (100 μmol/L). Twenty microlitre of Cell Counting Kit‐8 (CCK‐8) reaction solution was added to each well and incubated at 37°C for 1 hours in the dark. Absorbance of each well was measured by the optical density value at 450 nm.

### 2.7 Scratch wound assay

We scratched a linear wound through cells in a 35‐mm cell culture dish with a pipette tip and rinsed three times with 1 × PBS to remove cell debris. The culture medium was then replaced by media containing different concentrations of TGF‐β1 to investigate its effects on chondrocyte migration. Images were captured at 0, 12 and 24 hours following scraping. The mobility ratio was evaluated by dividing the area of the migrated cell region by area of the scraped region.

### 2.8 Scrape loading and dye transfer

The functional gap junctions between chondrocytes were evaluated with a scrape loading and Lucifer yellow transfer assay. Briefly, cells were seeded in dishes and grown to confluency. The medium was discarded, and cells were rinsed with CaMg‐PBS three times. A 1 mg/mL solution of Lucifer yellow dye (L0259; Sigma) was added to cover the cell monolayer, and the cells were scraped using a surgical blade. After a 5‐10‐minute incubation at room temperature, the cells were washed thoroughly with PBS and fixed by adding 4% paraformaldehyde. The cells were imaged with CLSM (Olympus & Nikon) to determine the number of fluorescent cells with Lucifer yellow uptake which is a measure of gap junction intercellular communication (GJIC).

### 2.9 Statistical analysis

All results are representative of at least three independent experiments. Statistical analysis was performed with SigmaStat 3.5 (Systat Software, Chicago, IL, USA) using one‐way ANOVA to compare the means of all groups. Data are presented as the mean ± SEM. Significant differences were defined as *P* < 0.05.

## 3 RESULTS

### 3.1 Expression of the TGF‐β super family genes in chondrocytes

**Figure 1:**
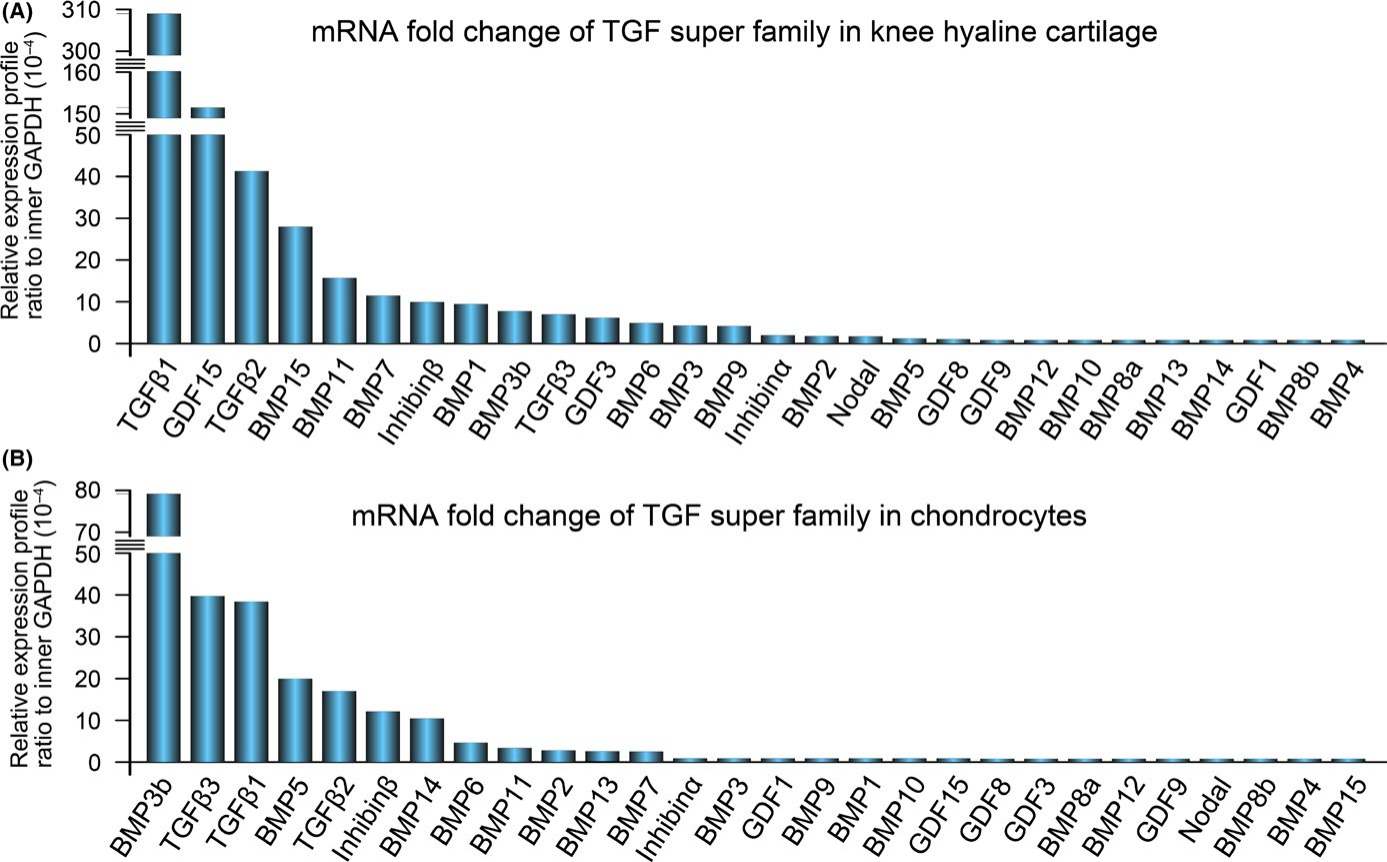
Expression of the TGF‐β super family in knee hyaline cartilage tissue and chondrocytes. A, qPCR confirmed gene profiles of the TGF‐β super family members in mouse knee hyaline cartilage tissue. The gene profiles of the TGF‐β super family were presented as the fold change ratio to the internal GAPDH control. B, qPCR confirmed gene profiles of the TGF‐β super family in mouse chondrocytes. The gene profiles of TGF‐β super family members were presented as the fold change ratio to internal GAPDH control. The fold change values were displayed in descending order. Data shown are representative of three independent experiments (n = 3). qPCR, quantitative real‐time PCR; TGF‐β, Transforming growth factor‐β1

The TGF‐β super family contains many secreted and structurally related proteins. The 33 members in the mammalian genome, including the TGF‐β isoforms, activins and inhibins, GDFs and BMPs^16^ were assessed in the current study. Based on our mRNA microarray data, we found that 28 members were expressed in chondrocytes. To confirm the mRNA expression levels of these genes, qPCR was used. The expression levels of 28 members in knee articular cartilage in vivo (Figure 1A) and primary chondrocytes in vitro (Figure 1B) are shown in descending order. The results indicated that TGF‐β1 mRNA was the most abundant in cartilage (up to 310 × 10^−4^‐fold change compared to the internal GAPDH control, Figure 1A). Even in the isolated primary chondrocytes, TGF‐β1 expression was also ranked in the top three (40 × 10^−4^ ‐fold change compared to the internal GAPDH control, Figure 1B). TGF‐β isoforms, BMPs and inhibin β ranked in the top 10 most highly expressed mRNAs tested in both cells and tissue, with BMP3b (80 × 10^−4^‐fold change) having the highest expression in cells. The expression levels of the other family members were relatively low compared to the genes mentioned above.

### 3.2 TGF‐β1 promotes gap junction intercellular communication in chondrocytes

**Figure 2:**
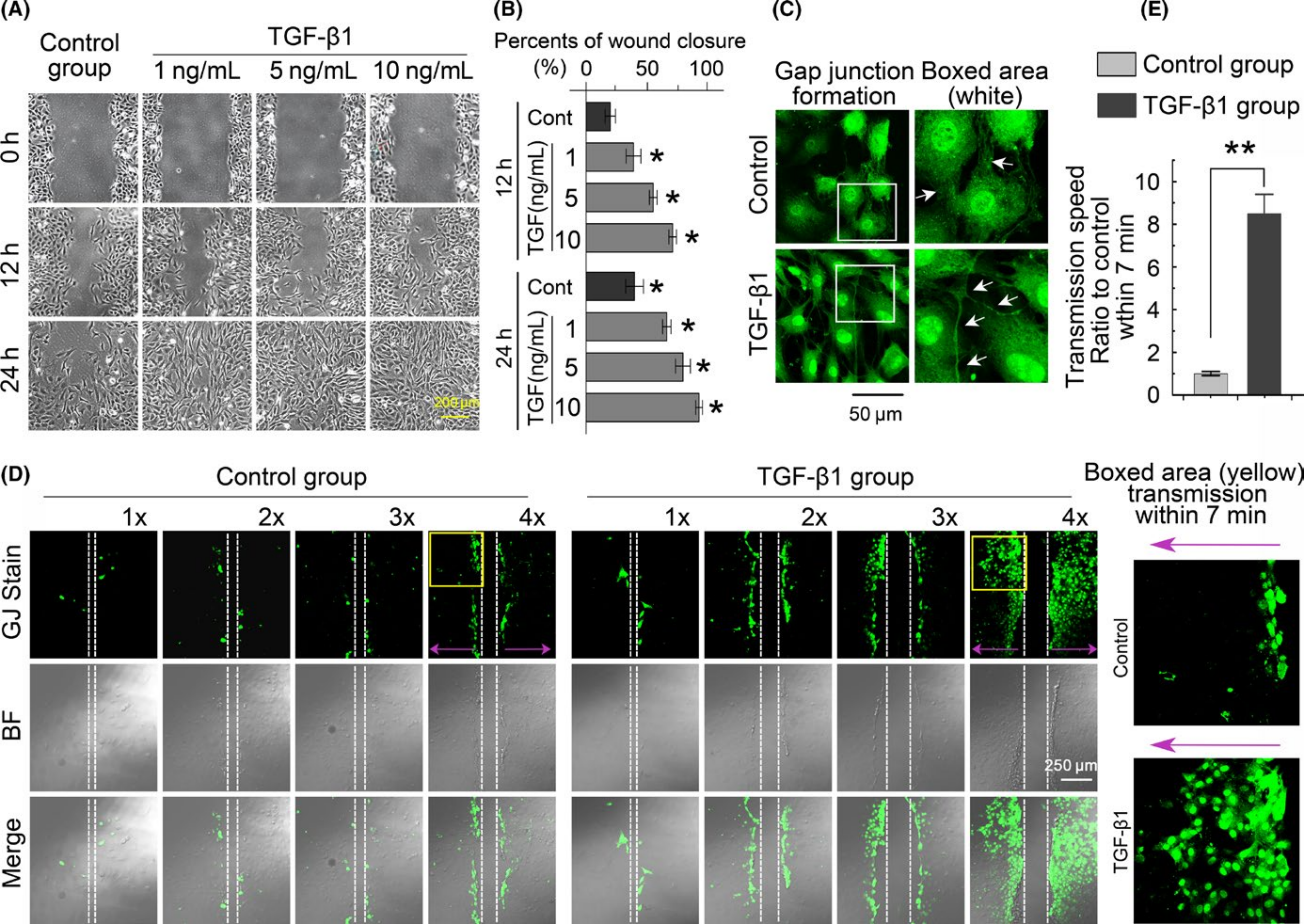
TGF‐β1 promotes gap junction intercellular communication in chondrocytes. A, Scratch assay showing that TGF‐β1 increased chondrocyte migration in a dose‐dependent manner. The images were based on three independent experiments (n = 3). B, Monolayer cells were scraped in the presence of 1 mg/mL of Lucifer yellow. LYdye assay (transferred for 30 minutes to trace functional cell‐cell connections among chondrocytes) showing functional gap junction formation was significantly enhanced in the TGF‐β1 (10 ng/mL)‐treated group relative to the control group. The white arrows denote long gap junction formations among chondrocytes induced by TGF‐β1. The images were based on three independent experiments (n = 3). C, The scrape loading/dye transfer (SL/DT) assay further demonstrated a cell density‐dependent increase in gap junctions in chondrocytes induced by TGF‐β1 (10 ng/mL). Images of chondrocyte cells positive for intercellular gap junction transfer as detected by Lucifer yellow dye in different phases of cell cultures experiments. The control groups refer to different densities of cells (1×: 2 × 10^5^ cells/mL; 2×: 4 × 10^5^ cells/mL; 3×: 6 × 10^5^ cells/mL; 4×: 8 × 10^5^ cells/mL). The TGF‐β1 group refers to the corresponding cell densities with 10 ng/mL TGF‐β1 treatment for 24 hours. The Lucifer yellow dye enters cells at the scratch (white dotted line) and is transferred to cells distant from the scratch (purple arrow). Intercellular gap junction transfer was calculated by measuring the distance from the scratching edge to the most distant cells with Lucifer yellow uptake. The images were based on three independent experiments observed with CLSM (n = 3). D, Quantification was performed to show different transmission speeds within 7 minutes of TGF‐β1 treatment compared to the control groups. Data are presented as the mean ± SEM (n = 3). ** *P* < 0.01. CLSM, confocal laser‐scanning microscopy; TGF‐β, Transforming growth factor‐β1

We first performed a scratch wound assay to determine the impact of TGF‐β1 on the migration and proliferation of chondrocytes (Figure 2A). The results showed that TGF‐β1 increased chondrocyte migration and proliferation in a dose‐dependent manner. The cell proliferation effect was further confirmed with the CCK8 assay (Figure S1). Gap junction formation, which occurs in the cell confluency process after migration and proliferation, was then assessed in the TGF‐β1‐pre‐incubated chondrocytes for 24 hours using Lucifer yellow dye (1 mg/mL). The chondrocytes extended long cytoplasmic cilia to connect to remote cells and form functional gap junction communications, which can transfer the Lucifer yellow small molecule from one cell to the others. The chondrocytes in the non‐treated control group just formed short wide extensions with neighbouring cells (Figure 2B). To further demonstrate whether the enhanced gap junctions induced by TGF‐β1 were functional, we performed the scrape loading/dye transfer assay. After 7 minutes of Lucifer yellow dye incorporation, the TGF‐β1‐treated group showed extensive Lucifer yellow transfer efficiency (Figure 2C). This increase in Lucifer yellow transfer efficiency was dose‐dependent (Figure S2). Moreover, the increase in Lucifer yellow transfer efficiency was cell density‐dependent in both the control and the TGF‐β1 group. The transmission speed of Lucifer yellow dye in the TGF‐β1 group was 8‐fold higher than that in the control group (Figure 2D).

### 3.3 TGF‐β1 promotes gap junction formation through increased expression of Cx43 in chondrocytes

**Figure 3:**
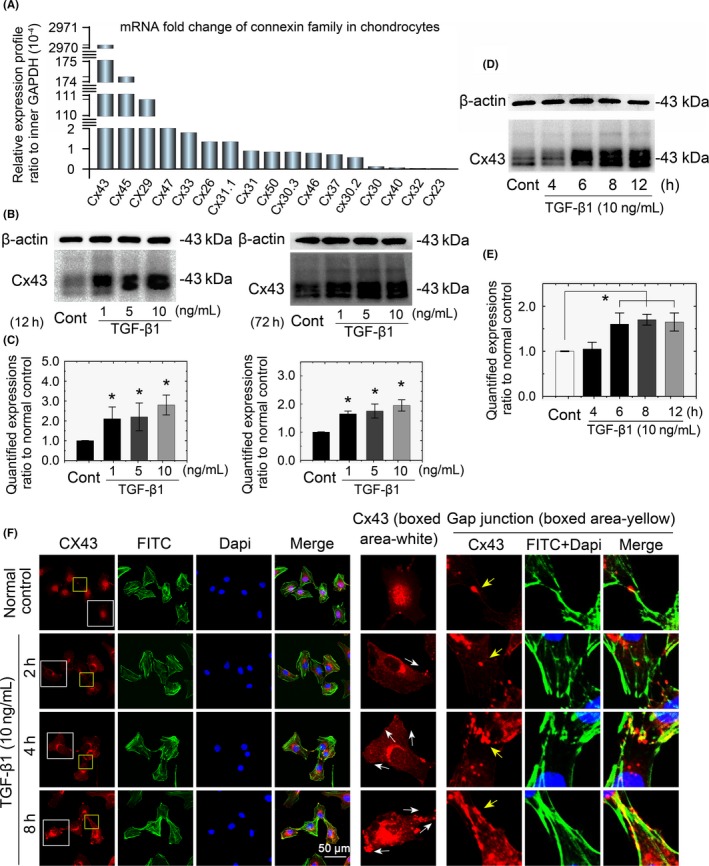
TGF‐β1 induced an increase in Cx43 in chondrocytes. mRNA microarray showing the gene profiles of the connexin family in chondrocytes. Seventeen connexin family members were detected in mouse chondrocytes. Primary chondrocytes isolated from articular cartilage were analysed. Data are presented as ratios to the internal GAPDH control. Fold change values were displayed in descending order. B, Western blots showing that TGF‐β1 promoted Cx43 expression in a dose‐dependent manner in early (12 hours) and late (72 hours) stages after treatment. The gels shown are representative of three different experiments (n = 3). C, Western blots showing that TGF‐β1 promoted Cx43 expression in a time‐dependent manner after the 10 ng/mL treatment. The gels shown are representative of three different experiments (n = 3). D, Immunofluorescence showing the TGF‐β1‐mediated change in localization of Cx43 in chondrocytes and gap junction sites between cells. Cytoskeleton stained with FITC‐ phalloidine (green) and nuclei stained with DAPI (blue). The TGF‐β1‐induced translocation of Cx43 from the nucleus towards the membrane border is indicated with white arrows (middle boxed area). The TGF‐β1‐induced accumulation of Cx43 at gap junction sites is indicated in yellow arrows (right boxed area). The images shown are representative of three different experiments (n = 3). Cx43, connexin43; TGF‐β, Transforming growth factor‐β1

Gap junctions consist of two paired hemichannels with connexin subunits.^17^ We found there were 17 connexin family members expressed in chondrocytes by mRNA microarray analysis (Figure 3A). The mRNA level of Cx43 was the highest (up to 2970 × 10^−4^‐fold change compared to the internal GAPDH control), followed by Cx45 (up to 174 × 10^−4^‐fold change), and Cx29 (up to 110 × 10^−4^‐fold change). The remaining connexin members all displayed a relatively low gene expression (<210 × 10^−4^‐fold change). To determine the effect of TGF‐β1 on Cx43 expression, the primary chondrocytes were treated with recombinant TGF‐β1 in different concentrations (1, 5 and 10 ng/mL). As shown in Figure 3B, the protein levels of Cx43 were significantly upregulated in a dose‐dependent manner both at the early stage (12 hours) and late stage (72 hours) as detected by Western blot assays (Figure 3B). Using 10 ng/mL TGF‐β1, we further showed that TGF‐β1 increased the expression of Cx43 in a time‐dependent manner (Figure 3C). Cx43 in primary chondrocytes showed multiple bands, representing different phosphorylation states of Cx43, with approximate molecular masses approximately 43 kDa, in agreement with the findings of several labs.^18^, ^19^

In untreated chondrocytes, Cx43 is primarily distributed to the nucleus. Cx43 translocation from the nucleus to the cytoplasm initiated 2 hours after TGF‐β1 induction. The prolonged accumulation of Cx43 in the cytoplasm was significantly enhanced 8 hours after TGF‐β1 induction (Figure 3D, Cx43‐boxed area in middle vertical lane). Additionally, in an analysis of cell‐cell connections (Figure 3D, gap junction‐boxed area in right vertical lane), we found that cells induced with TGF‐β1 had more Cx43 localized to cluster points along the cytoskeleton (F‐actin) between the two cells.

### 3.4 TGF‐β type I receptor is required for TGF‐β1‐modulated gap junction formation and Cx43 cytoplasmic translocation

**Figure 4:**
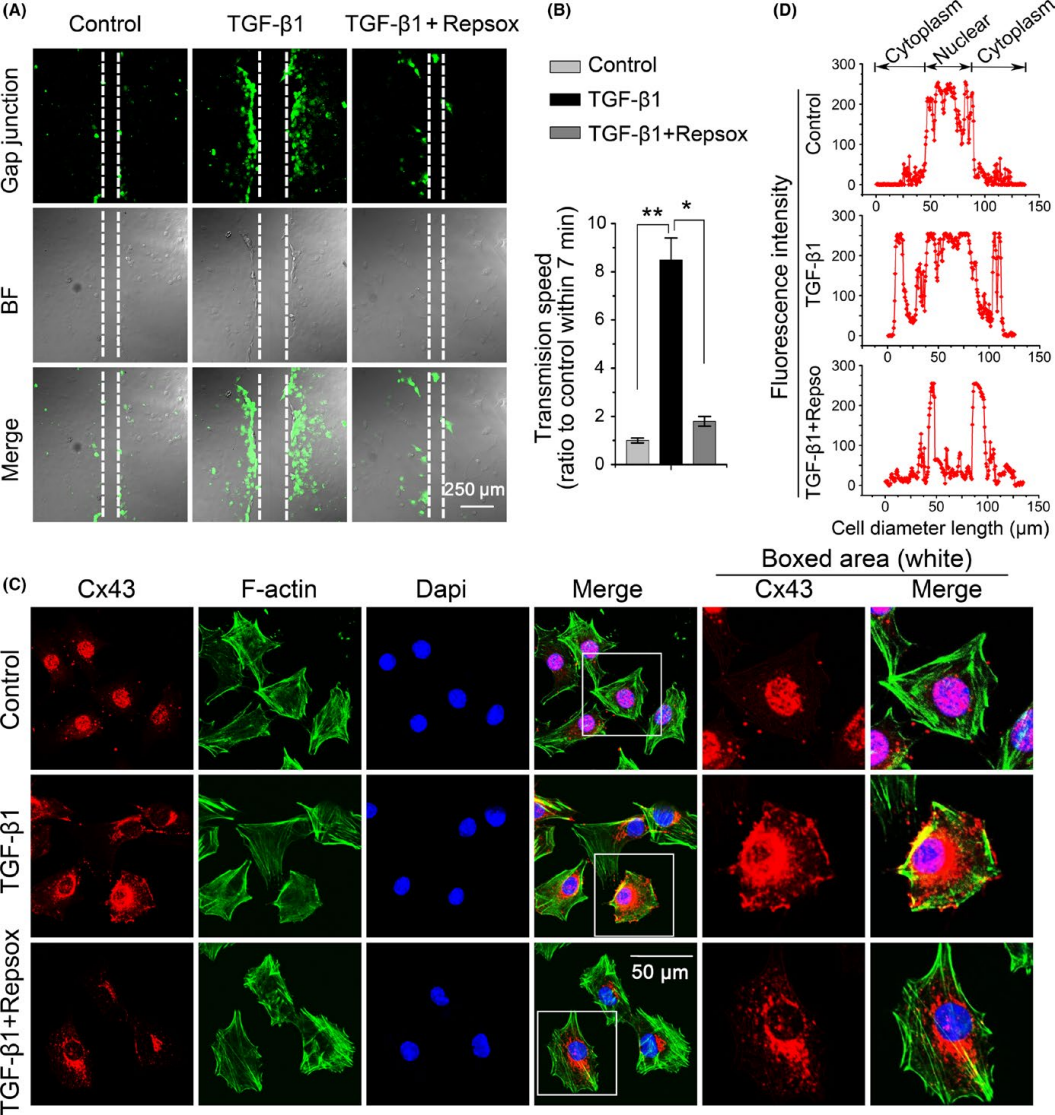
Repsox pre‐incubation attenuated TGF‐β1‐induced gap junction formation and Cx43 expression. A, Repsox pre‐incubation attenuated TGF‐β1‐induced gap junction formation. The concentration of TGF‐β1 was 10 ng/mL. The repsox used was at 100 μmol/L for a 12 hours pre‐incubation with primary chondrocytes. The images were based on three independent experiments observed with CLSM (n = 3). B, Quantitative analysis of the cell transmission speed of Lucifer yellow dye with respect to the control and TGF‐β1‐induced group (n = 3). *P < 0.05; **P < 0.01. C, Immunofluorescence staining of Cx43 (red) in chondrocytes after a 12‐hour treatment with TGF‐β1 (10 ng/mL) in the presence or absence of repsox (100 μmol/L). The chondrocytes were counterstained with markers of the nucleus (DAPI, blue) and actin cytoskeleton (phalloidin, green). The images were based on three independent experiments observed by CLSM (n = 3). D, Fluorescence optical density (OD) was analysed in Image Pro Plus 6.0 to measure the specific distribution of Cx43 cross the cell body. Data analysis was performed on at least 40 cells per group. CLSM, confocal laser‐scanning microscopy; Cx43, connexin43; TGF‐β, Transforming growth factor‐β1

Transforming growth factor‐β1 signal transduction mainly relies on TGF‐β type I receptor/ALK5 to activate the downstream signal of R‐Smad phosphorylation.^20^ To confirm the role of TGF‐β type I receptor (TβRI) in TGF‐β1‐induced gap junctions in chondrocytes, we conducted an inhibition experiment using a specific ALK5 inhibitor, repsox,^21^ to block TβRI. After pre‐incubated with repsox (100 μmol/L), the TGF‐β1‐induced formation of functional gap junctions was attenuated, as detected by reduced Lucifer yellow dye transfer speed (Figure 4A). The speed of Lucifer yellow uptake by chondrocytes was reduced to 21.1% within 7 minutes of inhibition (Figure 4B). Consistent with the above findings, TβRI inhibition disrupted the TGF‐β1‐induced distribution of Cx43. TGF‐β1‐treated chondrocytes had many Cx43 cluster points in the perinuclear region and at the edge of the cell membrane, but TβRI inhibition reduced Cx43 in the perinuclear region and eliminated its localization at the edge of the cell membrane (Figure 4C, boxed area). These results were further confirmed with a fluorescence optical density assay (Figure 4D).

### 3.5 TGF‐β1 modulates chondrocyte gap junctions through potential binding sites of downstream Smad signalling molecules in the promoter of Cx43

**Figure 5:**
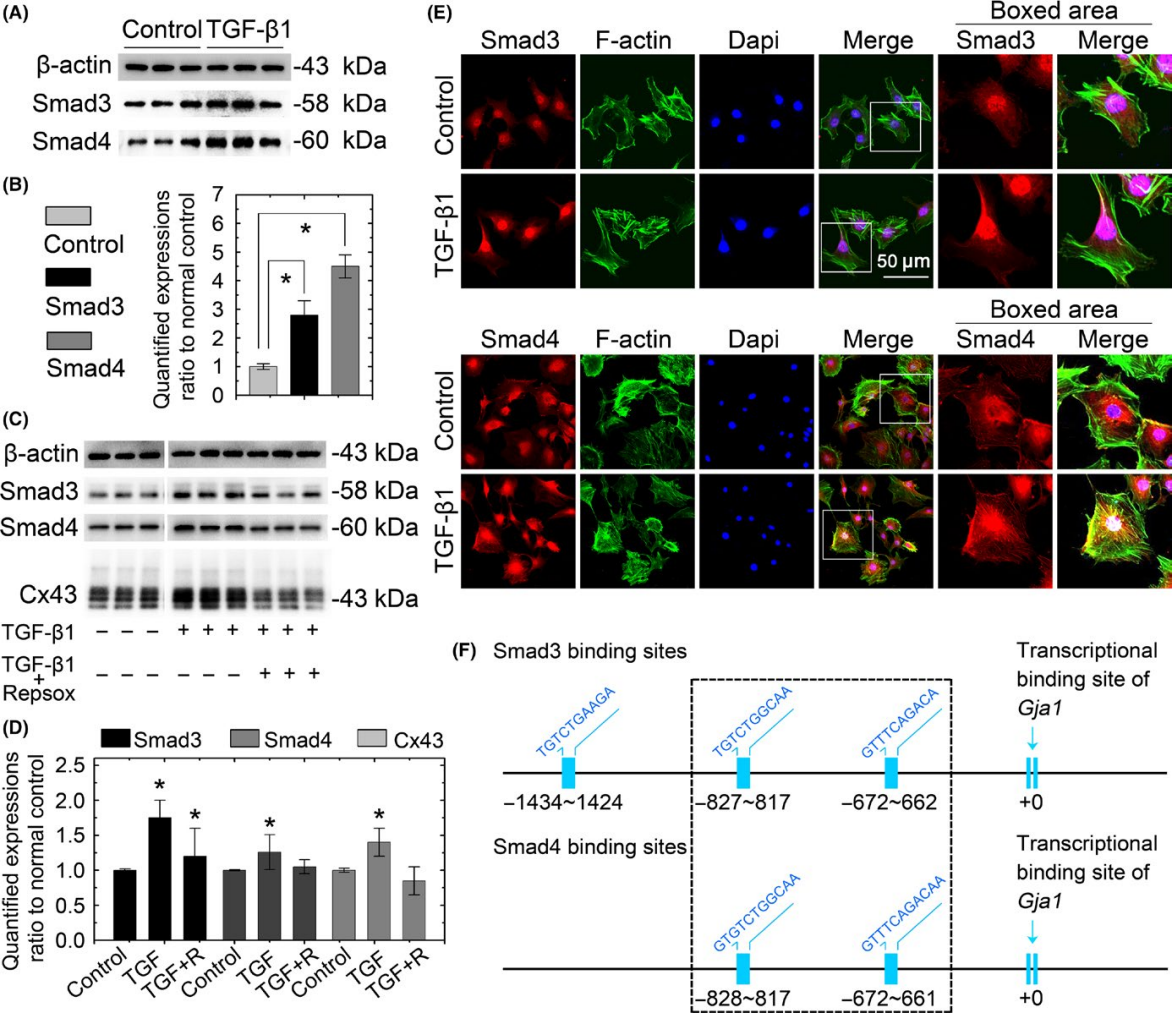
TGF‐β1 modulates expression of Cx43 in chondrocytes via Smad3 and Smad4 signalling. A, Western blots showing that TGF‐β1 increased the expressions of Smad3 and Smad4 in chondrocytes. Cell lysates were collected after a 2‐hour treatment with TGF‐β1 (10 ng/ml). The gels shown are representative of three independent experiments (n = 3). B, Quantification was performed to analyse the changes of Smad3 and Smad4 in (A). *Significant difference with respect to the untreated control chondrocytes (*P* < 0.05). C, Western blots showing that the repsox pre‐incubation attenuated the Smad3, Smad4 and Cx43 induction caused by TGF‐β1. Lysates were collected after 12‐hour TGF‐β1 (10 ng/mL) treatment with or without a repsox (100 μmol/L) pre‐incubation. The gels shown are representative of three independent experiments (n = 3). D, Immunofluorescent images showing the translocation of Smad3 and Smad4 in chondrocytes after induction with 10 ng/mL TGF‐β1. The chondrocytes were counterstained with markers of the nucleus (DAPI, blue) and actin cytoskeleton (phalloidin, green). The images are based on three independent experiments observed by CLSM (n = 3). E, Bioinformatics analysis by PROMO resource showing the putative Smad3‐ and Smad4‐binding sites in the promoter of Cx43 (*Gja1*, GeneBank name). Smad3 had three potential binding sites in the promoter of *Gja1*, and those sites were located at 1434‐1424 bp, 827‐817 bp and 672‐622 bp upstream of the *Gja1* transcriptional start site. Smad4 had two binding sites in the *Gja1* promoter located 828‐817 bp and 672‐621 bp upstream of the *Gja1* TSS. CLSM, confocal laser‐scanning microscopy; Cx43, connexin43; TGF‐β, Transforming growth factor‐β1

Following TGF‐β type I receptor activation, we aimed to elucidate the inner mechanism of TGF‐β1‐modulated gap junction formation in chondrocytes. We first showed that TGF‐β1 induced the expression of Smad3 and Smad4 by Western blotting (Figure 5A), and densitometry confirmed the increase (Figure 5B). With the TβRI inhibitor, we next found that repsox reduced the expressions of Smad3, Smad4 and Cx43 (Figure 5C). This synchronous change in Smad signalling components and Cx43 led us to consider their interactions. We found that TGF‐β1 induces Smad3 and Smad4 accumulation in chondrocyte nuclei by CLSM (Figure 5D). Using bioinformatics, we found a potential link between Smad3/4 nuclear translocation and the *Gja1* gene which encodes Cx43. We found three Smad3‐binding sites and two Smad4‐binding sites in the promoter of *Gja1* in the 2000 bp upstream of the transcription start site (TSS) (Figure 5E). Importantly, the Smad4‐binding sites were shared with Smad3 (same location and same sequences). The results indicated that Smad3 and Smad4, which can form a protein complex in the cytoplasm,^22^ translocate into the nucleus to synchronously initiate *Gja1* expression at the same binding sites.

## 4 DISCUSSION

Homeostasis of the articular cartilage is related to proper intercellular communication and function of the joined cells and relies on signalling molecules that pass between cell‐to‐cell and cell‐to‐ECM junctions. Connexin‐mediated GJIC is crucial for cell communication and cartilage function. TGF‐β1, as an indispensable growth factors in the cell life cycle, regulates Cx43 expression in a cell type‐dependent manner.^23^, ^24^, ^25^, ^26^ However, the effect of TGF‐β1 on Cx43 expression and the related GJIC in chondrocytes remains unclear.

The present study revealed for the first time that TGF‐β1 stimulation increased intercellular connections between chondrocytes containing Cx43 gap junction channels and hemichannels. Connexin proteins are subunits of gap junctions and hemichannels that accumulate on the plasma membrane of adjacent cells. When cells express multiple connexins, the connexins may co‐oligomerize into homomeric‐ or heteromeric‐connexins, although only certain combinations are permitted.^17^ Little is known about connexins in cartilage, but Cx43 was reported to be a highly expressed connexin in chondrocyte cells, while Cx45, Cx32 and Cx46 have also been detected.^27^, ^28^, ^29^, ^30^ In this study, we screened the connexin family and found that 17 connexin genes are expressed in mouse chondrocyte cells, with Cx43 being the most abundant.

A more detailed examination by confocal microscopy showed, in addition to the upregulation of Cx43 expression levels, TGF‐β1 treatment altered the Cx43 distribution in sub‐cellular structures of chondrocyte cells. Our study detected a dynamic and time‐sensitive delivery of Cx43‐containing connexins. Under TGF‐β1 stimulation, changes in focal Cx43 patterns (Figure 3D) were observed as early as 2 after treatment. Initially, Cx43 foci accumulated outside of the nucleus and then translocated from cytoplasmic structures to the cell membrane and finally into the intermembrane cytoplasmic area between cells with protruding lamellipodia ready to dock onto the mature gap junction plaques in a time‐dependent manner. This dynamic localization change also confirmed the known lifecycle of Cx43; Cx43 is first synthesized and transferred through the endoplasmic reticulum, and then it is oligomerized into connexin hemichannels in the trans‐Golgi network, and finally, it is laterally accreted from the plasma membrane into the gap junctions with increasing abundance over time (Figure 3D yellow arrow) in accordance with previous studies.^31^, ^32^ In the present study, cell proliferation was enhanced as TGF‐β1 stimulated the Cx43 expression pattern. We speculate that besides GJ formation, Cx43 may have a dual role in controlling cell proliferation. Lamiche et al^33^ connected the divergent Cx43 functions with differential localizations at either the membrane or the cytoplasm, as examined by cell proliferation assays. Specifically, the C‐terminal tail of Cx43 contains a nuclear targeting sequence that can directly mediate gene transcription of cell growth and cell death regulators.^34^, ^35^ Further studies are required to test this hypothesis.

Chondrocytes are the only cells found in healthy knee hyaline cartilage. Chondrocytes are found in small lacunae isolated from each other, and diffusion of substances may be the primary method of communication.^36^ The present results show that under monoculture conditions, chondrocytes connect directly with each other by forming gap junctions, which was confirmed by LY uptake (Figure 2B). Interestingly, TGF‐β1 induced morphological changes in chondrocytes resulting in an elongated shaped with long thin cytoplasmic projection arms extending to reach remote cells and forming a network structure of functional gap junctions which can transfer LY to distant cells. Previous studies reported similar cytoskeletal organization of extended cytoplasmic structures in chondrocytes with conflicting functions.^37^, ^38^, ^39^, ^40^ In this study, the elongated cellular projections formed between chondrocyte cells function as gap junctions, and this agrees with the spotted Cx43 plaques detected on cytoplasmic projection connection region between chondrocyte cells (Figure 3D).

**Figure 6:**
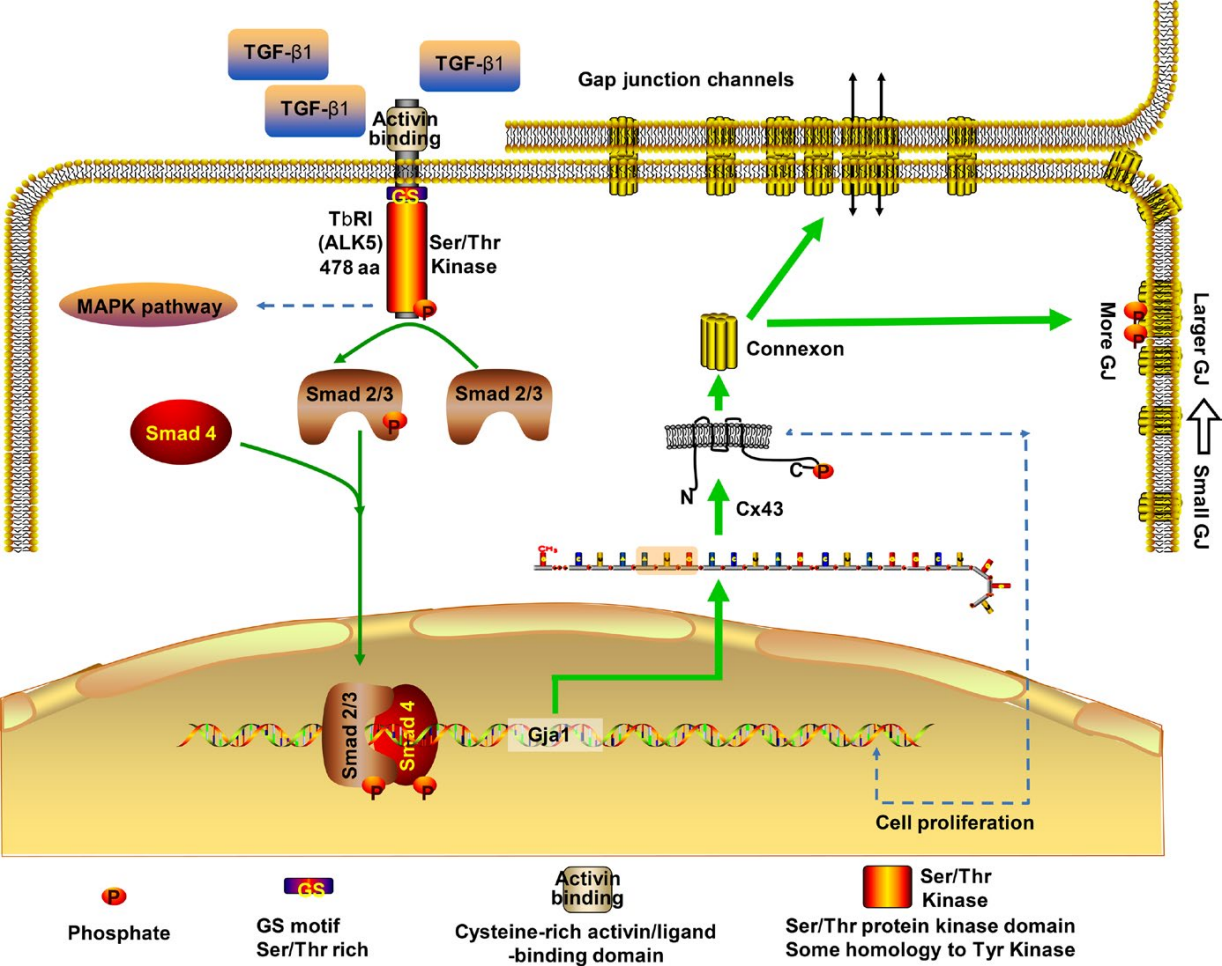
Schematic diagram illustrates the mechanism of TGF‐β1 regulation of Cx43 in chondrocytes. The green lines point to Smad‐dependent pathways elucidated by the current study, and the blue dotted lines are potential mechanisms not included in the present study. TGF‐β1 induced the canonical Smad signalling pathway, the activated TβRI phosphorylates the R‐Smads (Smad2, Smad3), transform them into transcriptional co‐regulator together with Smad4, and dock to potential binding site of promoter of Gja1 which encodes Cx43. As a result, Cx43 accumulated more on cell border and phosphorylated into functional gap junctions with larger size. Cx43, connexin43; TGF‐β, Transforming growth factor‐β1

To understand the potential mechanism of TGF‐β‐induced Cx43 regulation, we examined possible signalling molecules in the TGF‐β1 pathway. TGF‐β1‐mediated signalling is transduced at the surface through a complex of type I (TβRI) and type II (TβRII) receptors, which phosphorylate receptor‐activated Smads (R‐Smads) 3 and 4 and initiate Smad translocation into the nucleus where they ultimately regulate target genes such as transcriptional activators.^22^ To date, besides the canonical Smad‐dependent pathway, TGF‐β signalling also participates in a Smad‐independent signalling pathway that includes various branches of the mitogen‐activated protein kinase (MAPK) pathway.^20^, ^41^ In the present study, pre‐treatment with a strong TβRI/ALK5 inhibitor abolished the effect of TGF‐β1 on Cx43 upregulation and gap junction formation, indicating the involvement of the ALK5 receptor. TGF‐β1 significantly upregulated the expression of Smad3 and Smad4 as confirmed by Western blot analysis. In addition, TGF‐β1 stimulation also affected the sub‐cellular localization of Smad; immunofluorescence analysis detected Smad3 and Smad4 accumulation in the nucleus. Interesting, Smad4 seemed to co‐localize the cytoskeleton. Further, a bioinformatics database revealed that Smad3 and Smad4 occupy two identical binding sites in the promoter of the Gja1 gene, and this binding may promote Cx43 expression in accordance with TGF‐β1‐induced Cx43 expression in chondrocytes. Thus, it is possible that TGF‐β1‐mediated Cx43 signalling occurs via the canonical Smad pathway. The mechanism of this pathway is illustrated in Figure 6.

In conclusion, the current study revealed the molecular mechanisms of TGF‐β1‐mediated regulation of Cx43 in chondrocytes and confirmed that TGF‐β1 has a key role in cell communication, morphology and proliferation. The present results indicate these biofunctional roles of Cx43 are possibly induced by the TGF‐β type I receptor/ALK5‐driven canonical Smad3/Smad4 signalling pathway. Further studies to confirm the gene activation will be summarized in our next study. This study deepened our understanding of the molecular mechanisms that contribute to cell communication and Cx43 expression in chondrocytes.

## CONFLICT OF INTEREST

The authors declare that no competing interests exist.

## AUTHOR CONTRIBUTIONS

Qingxuan Wang and Jing Xie designed the study; Qingxuan Wang, Chenchen Zhou and Linyi Cai performed experiments and collected data; Jing Xie, Xiaobing Li and Jing Zou analysed and interpreted the data; Qingxuan Wang, Jing Xie and Demao Zhang drafted the manuscript; Jing Xie and Wenli Lai critically revised and approved the final manuscript.
