# Supplementary material for: TGF‐β1 promotes gap junctions formation in chondrocytes via Smad3/Smad4 signalling
Source: Cell Prolif. 2018 Nov 15;52(2):e12544. doi: 10.1111/cpr.12544 (PMC6495951; doi:10.1111/cpr.12544)
Supplement: Supplementary file 1 [file CPR-52-e12544-s001.doc]

**Supplementary Figures**

Figure S1


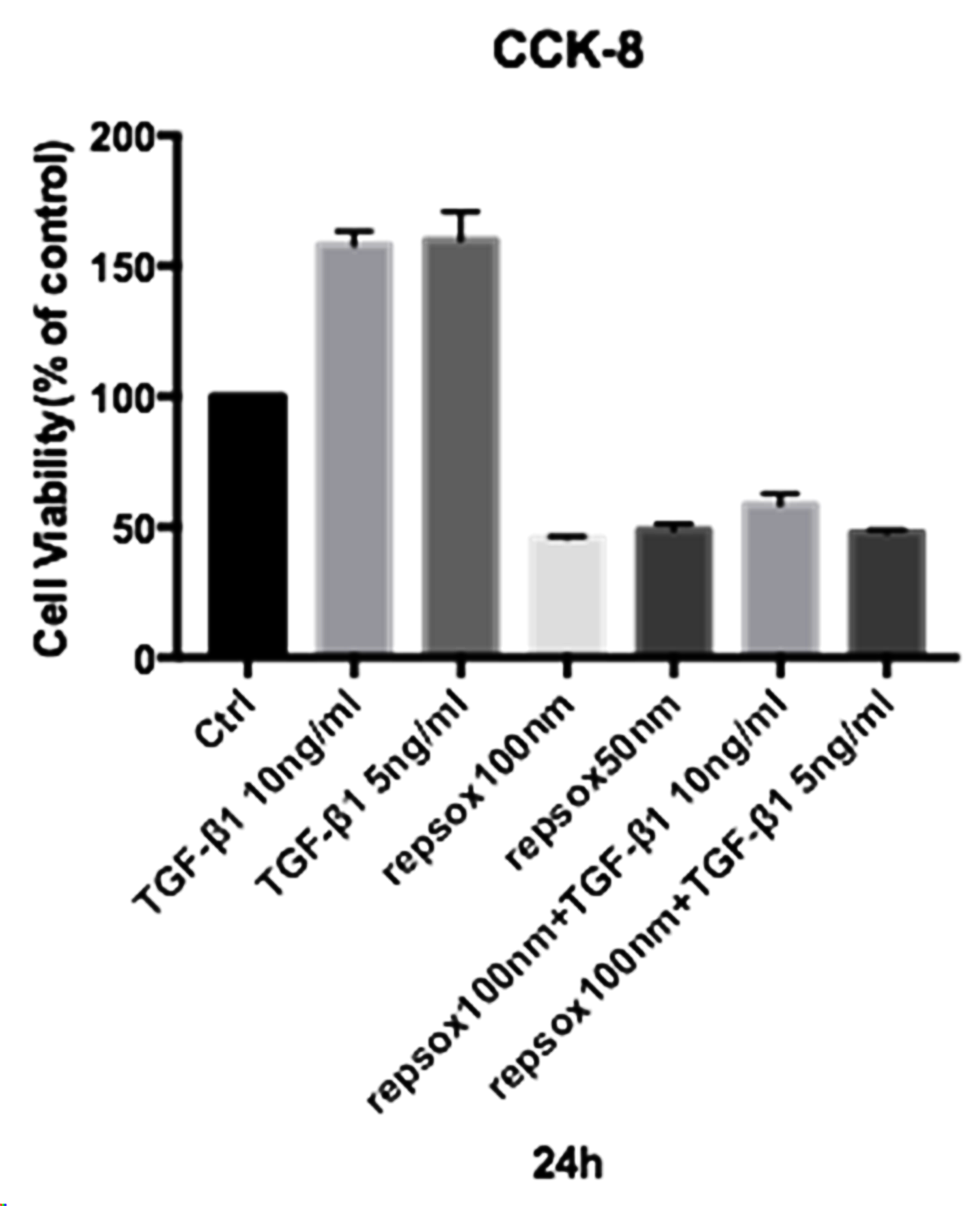


Fig. S1 The cell proliferation was performed by CCK8 asssay after treatment by TGF-β1, Repsox, and the combined two.

Figure S2


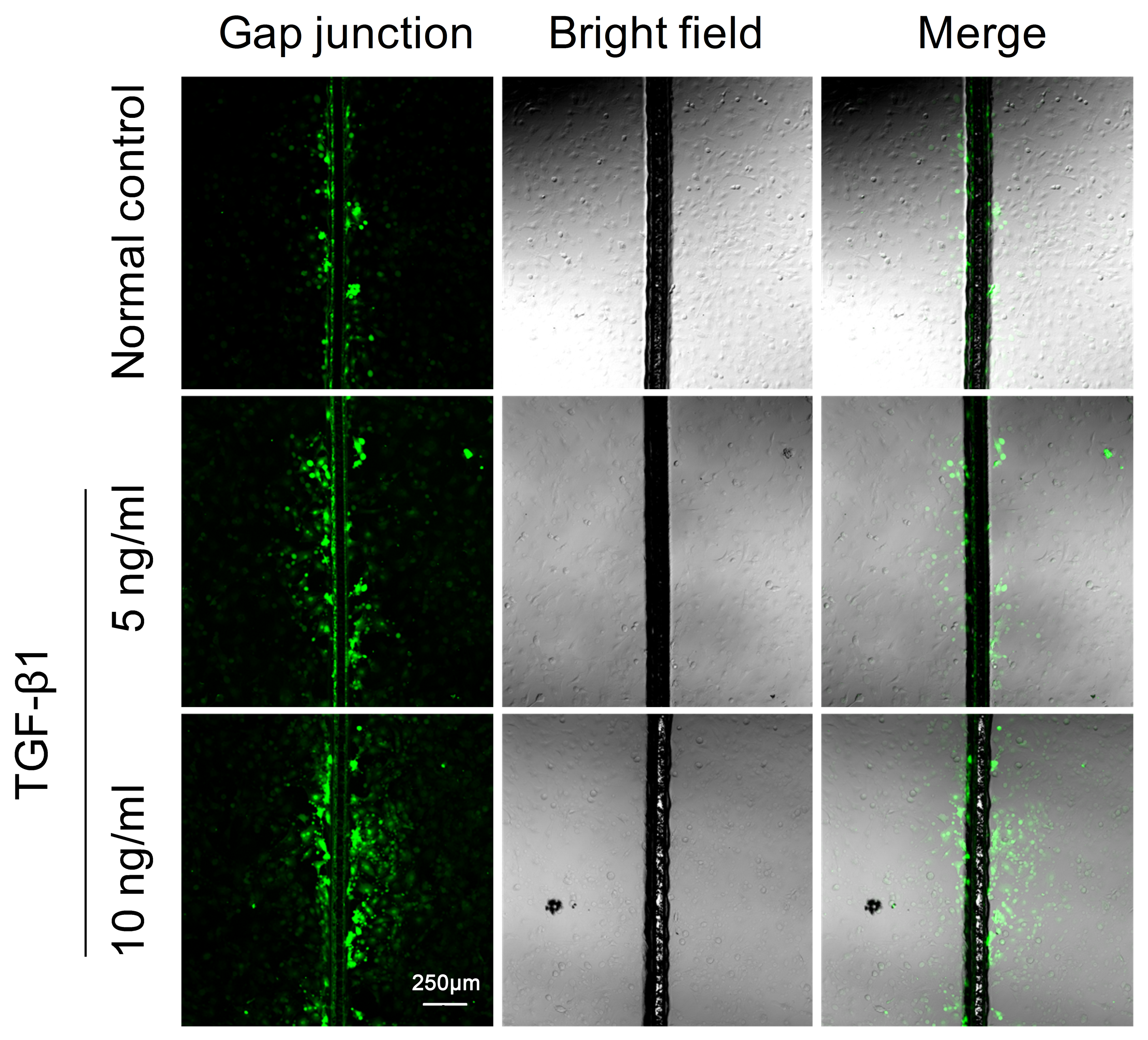


Fig.S2 TGF-β1 induced functional gap junction formation in a concentration-dependent manner in chondrocytes by Scrape Loading/LY Dye Transfer Assay. The images were obtained within 7 min after lucifer yellow dye staining.
